# Are GPs adequately equipped with the knowledge for educating and counseling of families with ADHD children?

**DOI:** 10.1186/1471-2296-11-5

**Published:** 2010-01-21

**Authors:** Ahmad Ghanizadeh, Nabi Zarei

**Affiliations:** 1 Research Center for Psychiatry and Behavioral Sciences, Shiraz University of Medical Sciences, Hafez Hospital, Shiraz, Iran; 2 Shiraz University of Medical Sciences, Hafez Hospital, Shiraz, Iran

## Abstract

**Background:**

Attention deficit hyperactivity disorder is one of the most common child psychiatry disorders. General physicians (GP), as primary care providers, can have an important role in screening and treatment of ADHD. This study aimed to survey GPs' knowledge, attitude, and their views of their role in the screening, diagnosing and managing children with ADHD.

**Methods:**

Six hundred and sixty five general physicians in Shiraz, Iran, answered a self-reported questionnaire on ADHD. The questionnaire consisted of questions regarding socio-demographic characteristics such as age, the duration of practice as a GP, marital status, general knowledge about ADHD, and the management of ADHD.

**Results:**

Less than half of them believed that they have adequate knowledge and information about this disorder. They usually do not like to be the primary care providers for children with ADHD. The majority of them prefer to refer the children to related specialists, mostly psychiatrists or psychologists. More than one third of them believed that sugar is a cause of ADHD. Only 6.6% of them reported that ADHD persists for the whole life. Their knowledge about methylphenidate is reasonable.

**Conclusions:**

As many other countries worldwide, the knowledge of GPs about ADHD should be improved. They do not asses and manage children with probable ADHD by themselves without referring to related professionals. They do not opt for the use of methylphenidate.

## Background

ADHD symptoms are very common among children and its rate in Iran was reported up to 10.1% [1]. Earlier diagnosis and management of ADHD is necessary and it affects the future of the children [2]. For example, a study reported that the majority of the school children with school refusal were suffering from ADHD [Ghanizadeh, Unpublished paper]. GPs can have an important and effective role in the diagnosis and management of ADHD. General physicians (GP) should have adequate knowledge to make probable diagnosis of behaviorals disorder such as ADHD [3]. However, their role is not clear and there are some controversies about it [4]. There are many significant barriers to be managed before implementing shared protocols for screening and management of ADHD [5]. One of the first steps is well educating of primary care physicians.

Already, the knowledge of GPs about intellectual and developmental disabilities has been studied in some countries such as Singapore, Pakistan, and Australia. They usually reported significant deficits due to inadequate training during medical education courses [4,6,7]. A study from Pakistan reported that GPs have inadequate knowledge about ADHD [4]. The study raised serious questions regarding the ability of GPs for the screening of ADHD at the primary care level [4]. A study on 156 pediatric residents in Turkey reported that 81.4% of them have inadequate knowledge on ADHD and 85.2% of them did not know the protocol for making diagnosis [8]. GPs in UK did not believe that they were equipped with adequate knowledge to diagnose or manag ADHD [5].

A previous study reported that GPs should be more informed about ADHD [9]. There was not found any published study about the knowledge of GPs regarding ADHD in Iran. However, there are similar studies on primary school teachers and pharmacists [10-12]. It is interesting that both of the two groups need to improve their knowledge about ADHD. Meanwhile, even parents of the children with ADHD have not enough knowledge about ADHD [9]. One of the reasons for delay in referring for diagnosis and management of children with probable ADHD is lack of knowledge of their parents about where or whom to refer to [9]. All of these findings show that children with ADHD are in the high risk of being undiagnosed and missed in Iran. In addition, there are a limited numbers of child psychiatrists in Iran. Even, many of the large cities might not have enough number of child psychiatrist. So, GPs as the primary care physicians or family physicians have a great responsibility and can have an important role in screening and diagnosis of ADHD. In addition, they are usually asked by the parents about the disorder, its related behaviors, diagnosis, and management including pharmacotherapy. This study aimed to research about the knowledge and attitude of the GPs towards ADHD and its management.

## Methods

This cross-sectional descriptive study included GPs from Shiraz, Southern Iran. About 900 GPs were registered in Shiraz Medical Council. 665 GPs privately answered the self-reported structured questionnaire to assess their knowledge, attitude, the source of acquired information about ADHD, and competence about the diagnosis and management of ADHD. The questionnaire was mailed to them and they were reminded to return their responses. The participants were provided with enough time to fill it, taking about 10 to 15 min. The questionnaires which were not returned at the time of gathering the data were not included in the analysis. The questionnaire had already been used in the previous studies on teachers and pharmacists [10,11]. Its validity and reliability were already reported [11]. Most of its statements are indicated in Table 1. It also contained questions about some of their demographic characteristics including age, the years of practice, and marital status. Another part of the questionnaire consisted of some true/false questions [4]. The subjects who did not answer a question were assumed to have no knowledge about the question.

**Table 1:** Knowledge and attitude of the general physicians about ADHD (n = 665)

Statements	%	Answer code*
ADHD have a biological and genetic predisposition	46.8	True
ADHD is not a serious problem and does not need to be managed.	19.5	True
ADHD can be caused by poor parenting practices and parental spoiling.	10.2	False
ADHD children are at a higher risk of truancy and escaping.	79.7	True
ADHD related difficulties are longlife.	6.6	True
ADHD children have a high risk for becoming delinquent as teenagers, substance use, and depression.	91.0	True
ADHD children's IQ is more than that of non-ADHD children.	24.8	-
ADHD children need psychological support.	96.8	True
Specially trained educators should teach these ADHD children.	68.9	True
The same discipline and rules used for all children should be applied to ADHD children	32.8	True
Educators should be aware of ADHD and ADHD children in the class.	92.6	True
ADHD children experience more difficulties in their relations with their classmates.	89.3	True
Educational achievement of ADHD children will be less than that of non-ADHD peers.	61.5	True
ADHD children experience more difficulties in their relations with their family members.	71.9	True
ADHD students should receive less homework than others.	35.8	True
ADHD can be treated and managed with proper medication.	74.6	True
ADHD can often be caused by sugar or food additives.	37.4	True
Chaotic and dysfunctional family is the etiology of ADHD.	52.3	True
Children with ADHD are misbehave primarily because they don't want to obey rules and do their assignments	82.3	True
Being able to watch TV or play with computer for minutes or hours rules out ADHD diagnosis	23.7	True

* The answer code may not represent the correct answer.

The questionnaire also asked their knowledge and attitude about methylphenidate and its use for ADHD management. Those questions were: "Is medication therapy necessary for treatment of ADHD? What is the clinical implication of methylphenidate? Which medication do you recommend as the best treatment for ADHD?" Which of the following medications (narcotics, sedatives, stimulants, and antipsychotic) does methylphenidate belong to? Do you agree that methylphenidate should not be administered in ADHD except in children with severe symptoms or specific difficult circumstances and that its prescription should be avoided?

The questionnaire was used in a pilot study on a small number of GPs to survey whether it can be administered easily and it has enough conceptual clarification. All of the participants were informed about the study and reassured that participation in the study is voluntary and anonymous. It was stressed that their responses would not be used for any other purpose. The study was approved by the office of chancellery for research affairs in Shiraz University of Medical Sciences.

Statistical analysis was conducted using SPSS-11. Frequencies were calculated for the demographic categorical data. The numerical values of age and the years of practice were reported as mean and standard deviation (SD).

## Results

### Demographic characteristics

665 questionnaires were returned. Excluding 316 (47.5%) of them who did not report their gender, 195 (55.6%) were male and 155 (44.4%) were female. The mean age of the whole sample was 39.3 (SD = 9.3) with a range of 24 to 80 years. 316 of them did not indicate their age. 73.2% of them were married. The mean years of their practice as a GP was 10.4 (SD = 8.5). Their familiarity with the statements associated with ADHD is displayed in Table 1, showing that nearly most of them were familiar with the statements. 20% reported that ADHD is not a serious problem. More than one third of them believed that sugar is a cause of ADHD. Nearly, 80% of them reported that ADHD is a risk factor for truancy and escape. Nearly, all of them stated that these children are at a higher risk of becoming delinquent as teenagers, substance use, and depression.

Just 3.8% of the GPs believed that the IQ of children with ADHD was lower than that of non-ADHD children. 68.6% of them mentioned that their IQ was similar to their peers and 24.8% responded that their IQ was more than that of their peers. Less than two thirds of them agreed that educational achievement of ADHD children is less than that of their peers. 6.6% reported that ADHD would persist for the whole life. 69.3% of the GPs indicated that Ritalin is a stimulant medication. 51.6% of the sample reported that Ritalin should not be administered for management of ADHD, unless in severe cases. 93.5% believed that Ritalin is used to increase attention and concentration.

Medical journals (n = 215, 32.3%) were the most common source of information. Media/magazines (n = 165) and colleagues/peers (n = 119) were the next common sources of their information. The minority of them (n = 65) have passed special courses on ADHD. About 46.6% of them believed that they have adequate knowledge and information about this disorder.

The GPs reported that ADHD should be managed by a psychiatrist (70.5%), psychologists (20.3%), neurologist (2.6%0, internist (2.4%), and other specialists (1.1%). Five percent of them asses and manage children with probable ADHD by their own without referring to experts. 44.5% of them refer these children without conducting any further assessment. 18.0% reported that they accept to manage them in follow ups.

## Discussion and Conclusions

As to the serious outcomes of ADHD, the most prominent finding is that many of the GPs have not sufficient information about at least some of the statements surveyed in the current study. According to a previous study in a clinical setting, less than 5% of the children with ADHD were suggested to be a possible case of ADHD for the first time by the GPs [9]. This may show that the GPs should be more equipped with some knowledge about AHD to be able to play a more active role in screening and treating it.

It is interesting that only 6.6% of the respondents agree that ADHD is a lifelong disorder. This rate is much lower than those reported in Turkey (35.2%) and Singapore (27%) [7,8]. Their response regarding the statement that ADHD is a lifelong disorder was very similar to those of the parents of children with ADHD. The rate for the parents was 6.0%. In other words, both of the parents and the GPs have a sense of optimism about this subject. About 46.9% reported that ADHD has a genetic predisposition while in the study in Turkey it was 63.4%.

However, near 80% of the GPs disagreed that it is not a serious problem and does not need to be managed. This rate is higher than that of 26.7% reported by the parents of children with ADHD [9]. About 37% of them believed that sugar was a reason for this problem which is much higher than the rate of 11.3% reported by the parents. Most of them reported that ADHD is a risk factor for truancy and escape which is much higher than the rate of 35.3% reported by parents. While 68.6% of the GPs mentioned that their IQ is similar to that of their peers, this rate for parents was 45.9%. Their knowledge about educational achievement of ADHD children is very similar to that of the parents (51.5%).

While most of them had a good knowledge about methylphenidate, half of them were against the use of methylphenidate for these children except for severe cases. It may indicate that their concerns about the use of the medication need to be considered in continued medical education (CME) programs. This gap has also been reported in a similar study on pharmacists [10].

The rates of the source of information were very similar to those of a similar study on GPs and pediatricians in Pakistan [4]. They reported that peers/colleagues and medical journals were the most common source of information. The rate of the claimed adequate knowledge was less than that reported in a study from Pakistan which was 64.4%. However, only 13.7% of the GPs from Pakistan had sufficient knowledge on effective screening/diagnosing of ADHD. Near 46.6% of the GPs in the current study claimed that they had adequate knowledge about the disorder.

The majority of the GPs reported that ADHD should be managed by a psychiatrist (70.5%) or psychologists (20.3%) and the GPs do not asses and manage children with probable ADHD by their own without referring to other experts. Our results regarding the necessity of management by psychiatrists/psychologists are very similar to those of the study from Pakistan, indicating that about 79% of them agreed with it [4]. The findings that about 75% of the GPs refer the probable cases of ADHD to specialists is in the same line with the study from Australia, revealing that GPs did not like to be the primary providers of care for children with ADHD [13]. They usually prefer to refer the patient to medical specialists. Perhaps, they are worried about over-diagnosis due to possible uncertainty for making decision about ADHD.

There are some limitations in the study that should be considered. Although the sample size was large, the answer of the questions was the binary response of false or true. So, the respondants had 50% chance to guess the correct answer.

Moreover, as much as ADHD is a common psychiatric disorder, it is a complex problem with many controversies, e.g. about three quarters of children with ADHD have at least one co-morbid psychiatric disorder [14]. Oppositional defiant disorder and learning disorder are the two common co-morbid disorders that should be differentiated from ADHD. In addition, there are some controversies and concerns about prescription of methylphenidate in ADHD. So, making decision on ADHD diagnosis and its management needs enough knowledge and expertise. Therefore, it might be an oversimplification to conclude that GPs have enough knowledge about the disorder.

### Implications

GPs as the primary care physicians should improve their knowledge regarding ADHD through participation in CME courses. Even, more emphasis is required on teaching and training regarding ADHD screening in undergraduate medical curricula. Meanwhile, for more training, GPs should be taught how to use structured diagnostic tools [4]. KSADS Farsi (Persian) version has been indicated to have enough sensitivity and reliability for making diagnosis [15]. Fortunately, GPs in Iran receive formal training in child and adult psychiatry in recent years. However, more education and training such as child psychiatry rotations might improve the current situation. Seminars and congresses on ADHD might be helpful. Further studies are required to evaluate the GPs' practices in making diagnosis and management of ADHD.

## Competing interests

The authors declare that they have no competing interests.

## Authors' contributions

A.G. conceived the study. A.G. and N.Z. jointly contributed to the study design. N.Z was responsible for gathering of the data. A.G. drafted the manuscript. A.G. and N.Z. performed the statistical analysis. All authors contributed to interpreting the results, and read and approved the final manuscript.

## Pre-publication history

The pre-publication history for this paper can be accessed here:

http://www.biomedcentral.com/1471-2296/11/5/prepub
